# Molecular Genetics of Renal Cell Carcinoma: A Narrative Review Focused on Clinical Relevance

**DOI:** 10.3390/curroncol32060359

**Published:** 2025-06-18

**Authors:** Braden Millan, Lauren Loebach, Ruben Blachman-Braun, Milan H. Patel, Jaskirat Saini, W. Marston Linehan, Mark W. Ball

**Affiliations:** Urologic Oncology Branch, National Cancer Institute, National Institutes of Health, Bethesda, MD 20892, USA; braden.millan@nih.gov (B.M.); lauren.loebach@nih.gov (L.L.); ruben.blachmanbraun@nih.gov (R.B.-B.); milan.patel@nih.gov (M.H.P.); jaskirat.saini@nih.gov (J.S.);

**Keywords:** molecular genetics, germline variants, somatic variants, circulating tumor DNA, renal cell carcinoma, next-generation sequencing, panel testing

## Abstract

Molecular testing in renal cell carcinoma (RCC) has allowed for a better understanding of the biology of both sporadic and hereditary diseases, where genetic testing is currently recommended in the guidelines for a select population with risk factors. Historically, screening, surveillance, and management decisions were based solely on clinicopathologic data; however, we now know that molecular profiling can enhance decision making, altering the treatment plan, approach, or selection of systemic therapy and enhancing the delivery of precision oncologic care. Advances and the increasing availability of next-generation sequencing technologies have improved the identification of germline and somatic variants in key RCC-associated genes. Given the molecular heterogeneity of RCC, these modern methods can identify unique genetic events that occur in a single individual, allowing for distinction between a metachronous tumor from metastases. Separate four-tier systems have been proposed to categorize germline and somatic variants according to their clinical significance, which should be highlighted. Additionally, emerging technologies, such as liquid biopsy, show potential for enhancing precision oncology in RCC. With this said, challenges, such as variant interpretation, ethical considerations, and accessibility, persist. This review examines the molecularly defined RCC, genetic testing methodologies currently available, their current clinical applications, limitations, and future directions.

## Simple Summary

The most common form of kidney cancer is renal cell carcinoma (RCC) which can run in families or occur by chance. Modern testing methods allow us to identify individuals who require close monitoring or early intervention, in addition to the ability to characterize the specific subtype of RCC, which allows for more personalized treatment. This review explains the latest methods used to test for these genetic changes, including new blood-based tests and advanced lab techniques. It also highlights the most common hereditary and molecular subtypes of RCC. While there are still challenges, such as cost and access to testing, this approach represents a major step toward personalized care in kidney cancer.

## 1. Introduction

Renal cell carcinoma (RCC) is the most common kidney malignancy, accounting for approximately 3% of all adult cancers worldwide, with increasing incidence and mortality over the past three decades [1,2]. RCC classically consists of three major histological subtypes: clear cell RCC (ccRCC), papillary RCC (pRCC), and chromophobe RCC (chRCC). Approximately 75% of cases are ccRCC, whereas pRCC and chRCC account for approximately 15% and 5% of cases, respectively [3]. While the majority of RCCs occur sporadically, an estimated 5–8% result from hereditary genetic variants, warranting screening for high-risk individuals. By studying hereditary RCC syndromes, many of the causal pathologic variants have been identified and studied, and we now know that many of these variants are detected in sporadic RCC [4,5,6]. To date, there have been at least 19 different genes shown to be associated with the development of RCC [7,8,9]. Given the advancements and understanding of the role genetic variants play in RCC pathophysiology, the World Health Organization (WHO) updated its guidelines in 2022 to include molecularly defined renal tumors in addition to the historic morphology-based classification [10]. These include *TFE3*-rearranged RCC, *TFEB*-altered RCC, *ELOC* (elongin-C-mutated; formerly *TCEB1*)-mutated RCC, *Fumarate hydratase*–deficient RCC (FHdef-RCC), Hereditary Leiomyomatosis and RCC (HLRCC) syndrome, succinate-dehydrogenase-deficient RCC (*SDH*), *ALK* rearranged RCC, Medullary carcinoma (not otherwise specified [NOS]), and *SMARCB1*-deficient medullary, dedifferentiated, and undifferentiated RCC [10]. These complexities in the genetic, pathological, and clinical aspects of RCC warrant better understanding of the subtypes by clinicians and what molecular testing is available and appropriate to enhance precision oncology.

Molecular testing has become critical to RCC management, helping to differentiate between hereditary and sporadic cases while informing treatment decisions. The rapid expansion of molecular diagnostics has enabled the characterization of RCC at both the germline and somatic levels, influencing clinical decision making and guiding the use of targeted therapy and immunotherapy combinations for both localized and advanced disease states [11,12,13]. The current National Comprehensive Cancer Network^®^ (NCCN^®^) guidelines on kidney cancer recommend that a clinician consider genetic testing for individuals with a close blood relative (first- or second-degree) who carries a known pathogenic variant in a cancer susceptibility gene or those diagnosed with RCC who are ≤46 years old, have bilateral or multifocal tumors, or have a family history of RCC. Testing is also recommended for individuals whose tumors show specific histologic features associated with hereditary syndromes [e.g., HLRCC, Birt–Hogg–Dubé (BHD), and tuberous sclerosis complex (TSC)] [14]. In 2015, the American College of Medical Genetics and Genomics (ACMG) provided guidelines for referring patients to genetic counselors; however, in clinical practice, only about a quarter of patients meet the criteria for genetic testing [15,16]. Beyond germline testing, current guidelines do not endorse the use of other molecular testing modalities in clinical practice. Nevertheless, we can provide a precision approach to oncologic care through improved molecular characterization and understanding of the different forms of RCC. Genetic testing can help us make patient-specific surveillance and treatment recommendations based on the molecular profile, provided we understand the currently defined subtypes and the available genetic testing options [17]. This review highlights the current understanding of the molecular genetics of both sporadic and hereditary RCC, genetic testing methodologies in kidney cancer, and their clinical utility, challenges, and emerging advancements.

## 2. Genetic Landscape of Kidney Cancer

### 2.1. Genetic Variants in Sporadic Kidney Cancer

Sporadic RCC, which constitutes most cases, is now known to have a diverse array of genetic alterations that vary across histologic subtypes, contributing to its significant heterogeneity. In ccRCC, the most common subtype, the inactivation of the *VHL* gene on chromosome 3p, is a pivotal early event, occurring in up to 90% of sporadic RCCs. It is often accompanied by variants in chromatin remodeling genes, such as *PBRM1*, *SETD2*, and *BAP1*, along with cytogenetic alterations, including loss of 3p and gain of 5q [18]. Papillary RCC, the second most frequent form, is commonly associated with *MET* variants in up to 18% of sporadic cases [19]. Chromophobe RCC, though rarer, often displays widespread chromosomal losses (e.g., chromosomes 1, 2, 6, 10, and 17) and, occasionally, *TP53* variants [20]. Additionally, oncocytoma, a benign renal tumor with low malignant potential, has been shown to have somatic alterations in mitochondrial complex I genes, with a normal karyotype, although losses of 1p and Y may also be present [20]. Together, these insights underscore the limitations of our current knowledge on the complex genetic landscape of sporadic RCC, the limitations of our current understanding, and its critical implications for prognosis, therapy selection, and precision oncology.

### 2.2. Hereditary Kidney Cancer Syndromes

Several more commonly known hereditary syndromes are associated with RCC, each with distinct genetic and clinical characteristics. All have been used to further our understanding of the genetics of RCC, and, in addition to molecularly defined RCC, are highlighted in Table 1.

#### 2.2.1. Von Hippel–Lindau (VHL) Syndrome

VHL syndrome is an autosomal dominant disorder caused by variants in the *VHL* tumor suppressor gene. These variants lead to the aberrant stabilization of hypoxia-inducible factor (HIF), driving tumorigenesis [21]. Germline *VHL* variants are present in nearly all patients with VHL syndrome, making genetic testing a critical diagnostic tool [13]. At the cellular level, the *VHL* gene encodes the VHL protein, which forms a complex with Elongin B, Elongin C, and Cullin-2, which all interact to create the E3 ubiquitin ligase complex. Under normoxic conditions, HIF-α is hydroxylated on its proline residues by the enzyme PHD. This hydroxylated form of HIF-α is recognized by the VHL complex and ubiquitinated on its lysine residues, marking it for degradation through the proteasome [7,22]. In the case of VHL syndrome, a nonfunctioning VHL protein leads to impaired marking and degradation of HIF-α, which alters cell signaling as if the cell is in a hypoxic state. The accumulation of HIF-α in affected cells leads to the upregulation of several angiogenic, cell cycle, and proliferative pathways, including increased expression of VEGF, EGFR, EPO, TGF-α, and PDGF-β [23,24]. This is the driving force for many of the manifestations of VHL syndrome, including ccRCC, pheochromocytoma, CNS and retinal hemangioblastomas, endolymphatic sac tumors (ELST), and broad ligament/epididymal cystadenomas [25]. This pathway, connecting VHL’s role in oxygen sensing and HIF regulation, has proven vital for our understanding of tumorigenesis in sporadic RCC [18,26]. The screening and management of ccRCC in VHL syndrome is well-established, and it is recommended that all renal tumors be surgically managed once the largest reaches ~3 cm in size [27]. The introduction of the HIF-2α antagonist Belzutifan has reduced the frequency but not eliminated the need for surgical interventions in eligible patients and has the potential to decrease the cumulative lifetime surgical burden [13]. In VHL-associated ccRCC, long-term results from LITESPARK-004 show a sustained response to Belzutifan in patients with VHL, where 67% (95% CI, 54–79%) of patients with renal tumors achieved an objective response, paving the way for a new treatment paradigm for these patients [28].

#### 2.2.2. Hereditary Papillary Renal Carcinoma (HPRC)

HPRC is an autosomal dominant disorder characterized by activating variants in the *MET* gene, which promotes the development of bilateral, multifocal pRCC [29]. Additionally, a less common entity, biphasic squamoid alveolar papillary RCC (BSA-PRCC), has been described in a population of patients with a novel MET germline variant identified in France [30]. The MET receptor is expressed on the surface of renal epithelial cells and functions as a signal to initiate renal tubular repair following insults, such as ischemia or exposure to cytotoxic agents [31]. In HPRC syndrome, specific missense variants within the tyrosine kinase domain of the MET receptor result in its ability to auto-dimerize, auto-phosphorylate, and become constitutively activated, even in the absence of its typical ligand, hepatocyte growth factor. This abnormal signaling induces the unchecked upregulation of several downstream pathways that contribute to cell growth and differentiation, including the PI3K/AKT/mTOR, STAT, MEK/ERK, GRB2, GAB1, and RAC1 pathways, leading to tumorigenesis [32]. Identifying *MET* variants is particularly valuable for patient counseling and precision oncology, as current targeted therapies have shown limited efficacy in this patient population [12,33].

#### 2.2.3. Hereditary Leiomyomatosis and Renal Cell Carcinoma (HLRCC)

HLRCC is associated with variants in the *fumarate hydratase* (*FH*) gene, encoding the FH protein, where, following a second somatic alteration in the gene, the accumulation of fumarate results in a pro-oncogenic milieu, potentially leading to an aggressive pRCC [34,35]. *FH* variants drive the metabolism of affected cells toward aerobic glycolysis, rather than through the Krebs cycle and oxidative phosphorylation, to meet the metabolic demands of energy production. This shift in glucose metabolism to aerobic glycolysis in HLRCC tumors is a hallmark example of the Warburg Effect [36]. The resultant decreased FH enzyme activity leads to the accumulation of fumarate and the creation of a pseudo-hypoxic cellular environment. This accumulation of fumarate interferes with 2-oxoglutarate-dependent enzymes, inhibiting the enzyme PHD and resulting in a buildup of HIF-α and other metabolic disruptions [37]. The increase in aerobic glycolysis leads to a decrease in the activation of the cellular energy sensor, AMP Kinase (AMPK), which subsequently results in an increase in mTOR and fatty acid synthesis. The upregulation of HIF-α, its downstream target genes, and decreased AMPK result in a cascade of events leading to tumorigenesis [38]. *FH* loss also results in the activation of Nrf2-dependent antioxidant pathways, allowing for FH-deficient cells to be more resistant to reactive oxygen species [39,40]. Early detection through genetic testing is crucial for patient management, including lifelong surveillance and aggressive surgical management, either open partial nephrectomy with wide surgical margins or radical nephrectomy, based on the propensity for metastasis with small primary tumors [41]. Additionally, the efficacy of novel systemic therapy combinations for advanced disease underscores the importance of the underlying genetic variant that results in HLRCC-associated RCC [12].

#### 2.2.4. Birt–Hogg–Dubé (BHD) Syndrome

BHD Syndrome, caused by variants in *FLCN*, increases the risk of multiple subtypes of RCC, benign pulmonary cysts, cutaneous fibrofolliculomas, and colonic neoplasms [42]. These patients are at risk of developing hybrid and chromophobe RCC, where genetic testing facilitates differentiation from other hereditary RCC syndromes [43]. When variants disrupt FLCN, these cellular interactions are impaired, and the unregulated activity of TFE3/TFEB leads to unchecked cell growth, increased PGC1α-dependent mitochondrial biogenesis, and, ultimately, tumorigenesis [44]. BHD-associated RCC can manifest as bilateral, multifocal tumors with a penetrance of 29–34% at a median age of 48–51 years [42,45,46]. Unlike other hereditary RCC susceptibility syndromes, BHD showcases a spectrum of RCC histological subtypes that can even vary within the same kidney [43]. Approximately 50–67% of tumors are hybrid oncocytic–chromophobe, followed by chromophobe tumors (23–34%), clear cell (7–9%), oncocytomas (3–5%), and papillary (~2%) [47]. The more indolent nature of these tumors allows for a less aggressive surgical approach than other hereditary RCC-associated syndromes, allowing for maximal preservation of renal function [43].

#### 2.2.5. Tuberous Sclerosis Complex (TSC)

TSC is an autosomal dominant genetic syndrome caused by germline variants in the tumor suppressor genes *TSC1* and *TSC2*, which have important roles in oxygen, iron, energy, and nutrient-sensing pathways [48,49]. The *TSC1* gene encodes the protein hamartin, and *TSC2* encodes tuberin. Loss-of-function variants in *TSC1/2* promote tumorigenesis through the constitutive activation of the mTOR pathway. Clinical manifestations of TSC include skin angiofibromas, subependymal giant cell astrocytomas, cardiac rhabdomyomas, pulmonary lymphangioleiomyomatosis, and renal masses. Although benign renal angiomyolipomas and cysts are the most common renal tumors seen in TSC, 2–4% of affected patients can develop RCC [50]. Histologic subtypes of renal tumors in TSC include ccRCC, papillary architecture, prominent smooth muscle stroma, and hybrid oncocyte/chromophobe RCC [51,52]. Screening for renal masses is recommended every three years from childhood with contrast-based magnetic resonance imaging (MRI) [53]. Active surveillance for renal tumors in TSC is advised, with the thresholds for intervention typically being 3 cm for RCCs and 4 cm for angiomyolipomas. Selective angioembolization of large or symptomatic renal AMLs is an effective treatment for avoiding bleeding complications and preserving functional nephrons [54]. If surgical management is planned, tumor enucleation is the preferred approach for both AMLs and RCCs in TSC. Everolimus, an mTOR inhibitor, is also approved for the treatment of angiomyolipomas > 3 cm; however, tumor growth occurs upon treatment discontinuation [55,56].

#### 2.2.6. Succinate-Dehydrogenase-Deficient RCC

Germline variants in the enzyme succinate dehydrogenase (*SDH*), specifically the subunit SDHB, can cause a rare and aggressive form of RCC [57]. Variants in *SDH* impair the conversion of succinate to fumarate in mitochondrial metabolism, leading to a shift toward aerobic glycolysis through the Warburg Effect. The resulting pseudo-hypoxic environment resembles other molecular subtypes of RCC, where PHD enzyme inhibition and HIF-α induction contribute to a pro-oncogenic environment alongside other, less-understood mechanisms [58,59]. In addition to RCC, *SDH* variants are associated with hereditary pheochromocytoma and paraganglioma syndrome, as well as gastrointestinal stromal tumors [60]. While SDH-deficient RCC exhibits a diverse array of histological patterns, it is frequently characterized by a vacuolated eosinophilic cytoplasm [61]. Active surveillance of renal masses in patients with *SDH* germline variants is not recommended. Instead, we recommend partial nephrectomy with wide margins or radical nephrectomy if a solid renal tumor is identified, although current evidence remains limited.

#### 2.2.7. BRCA1-Associated Protein (BAP1)

BAP1 tumor predisposition syndrome (BAP1-TPDS) is an autosomal dominant disorder caused by germline alterations in the tumor suppressor gene *BAP1* [62]. *BAP1* encodes for a nuclear deubiquitinase that binds HCF-1 to regulate cell growth; alterations in the HCF-1 binding motif contribute to unregulated cell proliferation. Clinical manifestations of BAP1-TPDS include uveal melanoma, mesothelioma, cutaneous melanoma, atypical melanocytic tumors, hepatocellular carcinoma, cholangiocarcinoma, meningioma, and ccRCC [63,64,65]. *BAP1* alterations are observed in approximately 15% of sporadic and 100% of ccRCCs in patients with germline *BAP1* alterations, which are characterized by high-grade pathologic features, higher-stage disease, and earlier clinical onset [66]. Studies reported to date have demonstrated that BAP1-deficient ccRCC is an aggressive phenotype, and therefore surgery is recommended with either a partial nephrectomy with a wide margin or radical nephrectomy upon initial tumor detection rather than active surveillance [67]. Contrast-based MRI every one to two years starting at age 30 is recommended for screening, although data are currently limited to support this recommendation [68].

#### 2.2.8. Translocation RCC

Different subtypes of translocation RCC with heterogenous histology were formerly referred to as the MiT Family before subclassification by their molecular subtypes by the WHO in 2022 [10]. Xp11 translocation RCCs harbor gene fusions involving *TFE3*, whereas t(6;11) RCCs harbor gene fusions involving transcription factor EB (*TFEB*), which are less common [69]. The translocations occur with several different genes, creating chimeric proteins with different functions, which contribute to the variable phenotype of these variants [70,71]. There is a higher incidence in female patients, while the prognosis varies widely and appears to be related to the age of onset. Pediatric patients are more likely to have an indolent course in comparison to adults, who typically have aggressive disease with higher risks of metastases and cancer-specific mortality [72,73]. Given the possibility of late recurrence or the development of metastatic disease, long-term follow-up is recommended for all patients [74]. No known extra-renal manifestations exist in those with germline translocations in Xp11, and no specific screening protocol is currently recommended. Other gene translocations have been described, most commonly involving chromosome 3, resulting in a predisposition to RCC, including clear cell, papillary, and chromophobe histology [75].

#### 2.2.9. ELOC-Mutated RCC

ELOC-mutated RCC (the term mutated is used as this is the current definition in the WHO 2022 guidelines [10]) is a rare molecular subtype of RCC characterized by bi-allelic variants in the *ELOC* (formerly *TCEB1*) gene at 8q21.11 in the absence of *VHL* variants [26]. These patients also exhibit many of the same clinical manifestations of those with VHL syndrome, including pheochromocytoma, CNS and retinal hemangioblastomas, endolymphatic sac tumors (ELST), and broad ligament/epididymal cystadenomas [76]. It is now recognized as a unique diagnostic entity and more commonly associated with an indolent course, exhibiting longer progression-free and overall survival than stage-matched ccRCC, although cases of advanced/metastatic disease have been described [77]. A distinct cytogenetic signature for ELOC-RCCs has recently been described. It is characterized by a karyotype with few chromosomal copy number alterations other than monosomy 8 and an intact third chromosome [77,78,79]. Given the paucity of reported cases of this molecular subtype of RCC, there are no current recommendations for screening or management.

## 3. Molecular Testing Modalities

**Figure 1:**
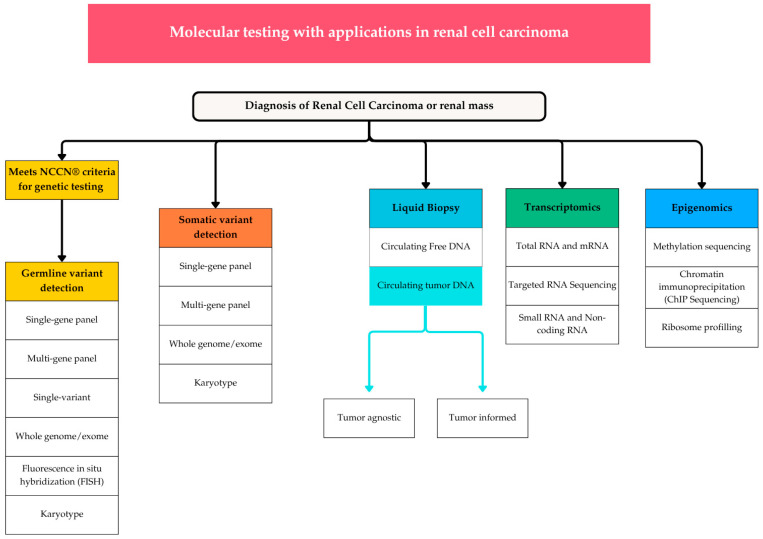
Molecular testing modalities with applications in kidney cancer. Germline genetic testing is the only current guideline-recommended investigation. NCCN^®^—National Comprehensive Cancer Network; DNA—deoxyribonucleic acid; RNA—ribonucleic acid; FISH—fluorescence in situ hybridization; ChIP—chromatin immunoprecipitation.

Historically, molecular testing modalities commonly available for cancer have included immunohistochemical (IHC) testing for the staining of specific proteins and fluorescence in situ hybridization (FISH) to detect chromosomal or transcriptomic abnormalities at the cellular level [80,81,82]. Molecular profiling using genetic testing for RCC includes germline and somatic genetic testing, liquid biopsy, transcriptomics, and epigenomics, each of which utilizes various sequencing techniques (Figure 1). Next-generation sequencing (NGS) allows for the detection of oncologic driver variants, including single-nucleotide variants, insertions, deletions, copy number alterations, fusions, tumor mutational burden, and microsatellite instability. Target enrichment or amplicon sequencing are techniques utilized to identify specific regions while eliminating regions that are not of interest [83]. Given its increasing availability (https://www.ncbi.nlm.nih.gov/gtr/ (accessed on 9 May 2025) and understanding of its clinical implications, oncologists in the United States are increasing the uptake of its use in practice [84]. Formalin fixation and paraffin embedding (FFPE) is the most widely used method for tissue fixation in pathology; therefore, high-throughput technologies have been adapted to overcome the challenges associated with the quality of DNA and RNA in FFPE tissue [85]. The current primary purpose of clinical NGS testing is to support decision making regarding disease pathogenesis or the selection of systemic therapy in advanced disease, although variant analysis is imperfect and needs to be interpreted with caution. If ordering these investigations, we must understand that the currently accepted terminology for alterations identified through genetic testing is the term “variant” with the following modifiers: (1) pathogenic, (2) likely pathogenic, (3) uncertain significance, (4) likely benign, or (5) benign [86].

### 3.1. Germline Genetic Testing

Given the complexity and implications of genetic testing, the systematic review by Bokkers and colleagues suggests that counseling patients can add up to 20 min to a consultation [87]. However, surgeons engaging their patients in a discussion about genetic testing can significantly increase their likelihood of undergoing testing, although, with increasing consultation requests, there is a shortage of genetic counselors [88,89]. Current American Society of Urologic Oncology (ASCO) guidelines recommend a conservative approach for patients who elect to undergo genetic testing. Germline genetic testing is recommended for those diagnosed with RCC before age 46, where the well-described hereditary cancer syndrome variants can be detected through either classical sequencing or NGS [90,91]. Single gene tests can target and identify alterations located in single genes known to cause a disorder and are often used to assess the family members of afflicted individuals in cascade testing. This approach enables various applications for discovering, validating, or screening genetic variants, including specific familial ones.

A limited multi-gene panel is recommended for patients with risk factors based on a personal or family history of malignancy, although the range in the number of genes per panel varies widely [92]. Mayo Clinic offers a pan-cancer NGS test, among others, for germline variants (mayocliniclabs.com), which includes sequence alterations in 515 genes, amplification of 96 genes, homozygous deletion of 133 genes, as well as detection of fusions involving 55 genes and complete inactivation of 31 tumor suppressor genes. Some of the other available multi-cancer tests offered by commercial companies include FoundationOne (foundationmedicine.com, 154 genes), nonAcus (nonacus.com, 146 genes), Illumina (www.illumina.com, 113 genes), Invitae (www.invitae.com, 70 genes), Myriad Genetics (myriad.com, 48 genes), and Ambry Genetics (www.ambrygen.com, 39 genes). Given these tests’ availability and low cost, this can allow for cascade testing for blood relatives of affected individuals with an identified hereditary cancer gene. Larger targeted panels may help identify patients with a higher rate of variants or novel cancer susceptibility genes [92]. Whole genome sequencing has identified millions of variants within humans, although the implications of the vast majority are still unknown [93]. In contrast, it is well-established that variants in exons, the protein-coding portion of the genome, are linked to the development of RCC and therefore should be the focus of any clinical investigation [49]. Whole genome/exome sequencing can provide a large amount of data and may allow for a more accurate estimation of the tumor mutational burden (TMB) in patients with resected solid tumors undergoing somatic genetic testing, which may have implications for response to systemic therapy in advanced disease [94].

### 3.2. Somatic Genetic Testing

Somatic testing detects tumor-specific variants that have the potential to influence treatment decisions, although it is associated with an increased risk of false positives due to the differences in allele ratios and tumor heterogeneity [95]. Current recommendations include offering germline genetic testing to patients in whom somatic testing from tumor tissue identifies specific variants; this should be discussed with patients before performing somatic NGS on the tumor specimen [92]. A different set of interpretation rules has been proposed for somatic NGS, as tumor-specific databases are often used in the absence of germline testing for individual patients. The standards and guidelines for somatic variants proposed by the Association for Molecular Pathology include a four-tier system. This includes tier I, variants with strong clinical significance (level A and B evidence); tier II, variants with potential clinical significance (level C or D evidence); tier III, variants with unknown clinical significance; and tier IV, benign or likely benign variants [86]. Additional benefits of somatic testing of solid tumors include the ability to identify metastases versus metachronous renal tumors [96]. As these assays become more commonly used in clinical practice, a framework will need to be developed to interpret clinically relevant variants. Some of the commercially available NGS tests for somatic variants using FFPE tissue include TruSight Oncology 500 (www.illumina.com, 500 genes), Foundationone^®^CDx (www.foundationmedicine.com, 324 genes), and xT CDx (www.tempus.com, 648 genes). Similarly to germline whole genome/exome sequencing, the results are challenging to apply clinically. As a result, targeted or amplicon-based testing is more commonly employed, as it focuses on specific regions of interest and allows for high-throughput testing of many amplicons simultaneously at a lower cost [85].

### 3.3. Cell-Free DNA (cfDNA) and Circulating Tumor DNA (ctDNA)

cfDNA is all of the extracellular DNA detected in the plasma, while ctDNA is the fraction that can be attributed to originating from cancer cells [97]. Assessment of cfDNA enables a non-invasive method for molecular profiling of patients with kidney cancer, and higher levels are associated with recurrence and metastases [98,99,100,101]. Efforts have been made to identify molecular predictors of response to systemic therapy in metastatic RCC, although challenges remain, as the concordance between variants identified using NGS of serum ctDNA and solid tumors was between 8.6% and 34.7% [102,103,104,105]. Furthermore, there are uniquely low levels of ctDNA in kidney cancer in comparison to all other extra-cranial malignancies [106]. Current evidence on the role of cfDNA and ctDNA is outlined in the critical review by Green et al. [107]. Some of the commercially available NGS tests for cfDNA and ctDNA include Guardant360 (www.guardantcomplete.com, 740 genes), FoundationOne^®^Liquid CDx (www.foundationmedicine.com, 309 genes), and Tempus xF+ (www.tempus.com, 523 genes). While these tests are currently available and used in many clinical trials, their use in clinical practice is poorly defined and not a part of guideline recommendations.

### 3.4. RNA Sequencing and Methylation Profiling

RNA sequencing (RNA-seq) and methylation profiling have emerged as pivotal tools in understanding the pathogenesis, tumor microenvironment, and therapeutic responses in ccRCC. RNA-seq, especially at the single-cell and spatial levels, allows for detailed characterization of the heterogeneous cellular composition of ccRCC tumors, enabling precise mapping of tumor-infiltrating immune cells and their transcriptional states [108]. Studies have identified 5-methylcytosine (m5C) RNA methylation regulators as critical players in tumor progression, immune cell distribution, and therapy response. These regulators can stratify patients into distinct prognostic subgroups with differing survival outcomes and immunotherapy susceptibilities [109]. Meanwhile, DNA methylation profiling complements RNA-seq by uncovering methylation-driven gene expression changes that contribute to oncogenesis and prognosis. Models based on methylation-regulated genes have demonstrated an association with overall survival and revealed genes like *NCKAP1L*, *EVI2A*, and *BATF* as prognostically significant [110]. Together, these omics approaches offer synergistic insights that inform personalized treatment strategies and biomarker development for RCC, although their current use in clinical practice is limited.

## 4. Discussion

This review examines the evolving role of molecular testing in renal cell carcinoma (RCC), emphasizing genetic testing and its relevance in both hereditary and sporadic disease. We outline novel insights into the molecular characterization of RCC syndromes, the expanding utility and limitations of current germline and somatic testing modalities, and the potential of emerging technologies, such as ctDNA, to inform personalized treatment strategies. Together, these advances underscore the shift toward a precision oncology framework in RCC.

Molecular profiling has become essential in managing hereditary RCC syndromes. While clinical features once guided surveillance and treatment decisions, gene-specific understanding enables more nuanced risk stratification and tailored management. For example, tumors associated with VHL syndrome can often be monitored until reaching a 3 cm threshold, whereas those associated with HLRCC, *SDHB*, and *BAP1* variants require early surgical management due to aggressive behavior [27,41,111]. These distinctions help bridge a clinical gap between quaternary and other centers, allowing clinicians to match management intensity with biologic risk and, ultimately, improve outcomes through individualized treatment.

Somatic and germline sequencing technologies are increasingly used in clinical practice for early diagnosis, screening protocols, treatment guidance, and even distinguishing metachronous tumors from metastases. However, real-world adoption remains uneven due to interpretive complexity, especially for variants of uncertain significance. Current frameworks, such as the NCCN^®^ and ACMG systems, offer guidance, but standardized reporting and clinician education are essential for broader integration [16,112]. Despite these challenges, somatic profiling holds promise for informing treatment selection and guiding genetic counseling referrals.

cfDNA and ctDNA are emerging tools that offer new opportunities to interrogate tumor heterogeneity, assess prognosis, risk stratify, and monitor therapeutic response. These modalities remain largely investigational in RCC, with technical limitations, such as low tumor shedding of DNA and variable concordance with tumor tissue, particularly relevant to ctDNA [107]. Nonetheless, the incorporation of multi-omic data has the potential to refine risk stratification models, guide patient management, and identify novel therapeutic targets. Ongoing clinical trials will be key to determining how these technologies can be effectively implemented in routine care.

There are several limitations to the current manuscript, and, as a narrative synthesis, it may be subject to selection bias regarding the articles included based on our center’s experience. Many diagnostic platforms discussed are validated in high-resource settings, limiting generalization. Additionally, insurance coverage and access to genetic counseling also remain barriers to equitable implementation. Moreover, while the biological rationale for many gene-specific interventions is strong, prospective clinical data remain limited, and most emerging technologies remain outside of clinical guidelines and require further validation.

Despite these limitations, this review highlights important complexities of hereditary and molecularly defined RCC, with a translational focus and synthesis of some of the available molecular tools, which are rapidly evolving. As advancements continue in the field, greater emphasis should be placed on genomic utility, including the value of genomic information in enhancing diagnostic accuracy, informing treatment and prevention strategies, supporting reproductive decision making, benefiting family members, and contributing to broader societal and healthcare outcomes [113]. Future directions include improving access to testing and counseling and generating prospective data to support gene-informed clinical decision making. Precision oncology in RCC is within reach, but realizing its full potential will require coordinated, multidisciplinary efforts.

## Figures and Tables

**Table 1:** Hereditary syndromes and molecularly defined renal cell carcinoma.

	Altered Gene	WHO/ISUP Tumor Classification	Screening Recommendations	Management	Type of Operation
VHL	*VHL*	Clear cell RCC	Contrast-enhanced cross-sectional imaging * every 2 years beginning at age 15 y	AS until largest tumor reaches 3 cm	Partial nephrectomy (enucleation) or ablative therapy ^+^
HPRC	*MET*	Papillary RCC (formerly type I papillary RCC), Biphasic Squamoid Alveolar Papillary RCC (BSA PRCC)	Contrast-enhanced cross-sectional imaging * every 1–2 years beginning at age 30 y	AS until the largest tumor reaches 3 cm	Partial nephrectomy (enucleation) or ablative therapy ^+^
HLRCC	*FH*	FH-deficient RCC (papillary); less common: chromophobe RCC	Contrast-enhanced cross-sectional imaging * annually beginning at age 8–10 y	Surgical management for any radiologically visible solid lesions ^&^	Open partial nephrectomy with wide margin, consider radical for large lesions, and retroperitoneal lymph node dissection for large and/or complex lesions
BHD	*FLCN*	Most common: hybrid oncocytic and chromophobe RCC; less common: clear cell, oncocytoma, papillary RCC (formerly type I papillary)	Contrast-enhanced cross-sectional imaging * every 3 years beginning at age 20 y	AS until the largest tumor reaches 3 cm	Partial nephrectomy (enucleation) or ablative therapy ^+^
TSC	*TSC1* or *TSC2*	Angiomyolipoma RCC: TSC-associated RCC with fibromyomatosis stroma, TSC-associated oncocytic tumor, eosinophilic solid and cystic tumor	Contrast-enhanced cross-sectional imaging * every 1–3 years beginning at age 12 y	AS until the largest tumor reaches 3 cm (RCC) and 4 cm (AML); angiography to determine the presence of intra-tumoral aneurysm	Tumor enucleation for RCC, selective angioembolization or tumor enucleation for AML or ablative therapy ^+^
SDH	*SDHAF2*, *SDHA*, *SDHB*, *SDHC*, *SDHD*	SDH-deficient RCC (diverse histology pattern)	Contrast-enhanced cross-sectional imaging * every 2 years beginning at age 12 y, concurrently with paraganglioma screening	Surgical management for any radiologically visible solid lesions ^&^	Partial nephrectomy with wide margin, consider radical for large lesions, open surgery for cystic lesions, retroperitoneal lymph node dissection for large and/or complex lesions
BAP1	*BAP1*	Clear cell RCC	Contrast-enhanced cross-sectional imaging * every 2 years beginning at age 30 y	Surgical management for any radiologically visible solid lesions	Partial nephrectomy (wedge) or radical nephrectomy
Translocation RCC	*TFE3*, *TFEB*, *MITF*, chromosome 3	Molecularly defined translocation RCC with features similar to clear cell RCC	No recommended screening protocol reported; long-term follow-up after diagnosis	Surgical management for any radiologically visible solid lesions	Partial nephrectomy (wedge) or radical nephrectomy
*ELOC*	Elongin C mutated (formerly *TCEB1*)	Molecularly defined RCC with features similar to clear cell RCC	No recommended screening protocol reported	No currently recommended approach	No currently recommended approach

Screening, management, and surgical recommendations per NCCN^®^ 2025 guidelines. Unless otherwise specified, robotic or open surgical approaches may be performed at the treating physician’s discretion. Acronyms: AML (angiomyolipoma), AS (active surveillance), *BAP1* (BRCA-associated protein 1), BHD (Birt–Hogg–Dubé), CNS (central nervous system), *ELOC* (elongin C), HLRCC (hereditary leiomyomatosis and renal cell cancer), HPRC (hereditary papillary renal cancer), MRI (magnetic resonance imaging), *MiTF* (melanocyte inducing transcription factor), RCC (renal cell carcinoma), SDH (succinate dehydrogenase), TSC (tuberous sclerosis complex), VHL (von Hippel–Lindau Syndrome), and y (year). * MRI preferred; renal ultrasound is generally not recommended for screening. ^&^ biopsies are typically avoided due to increased risk of tumor seeding. ^+^ option for those with significant medical or surgical risk to undergo an operation.
